# Benchmarking General Purpose Artificial Intelligence for Accessory Pathway Localisation on 12-Lead Electrocardiograms: A Proof-of-Concept Study

**DOI:** 10.3390/jcm15114058

**Published:** 2026-05-24

**Authors:** Ahmed Abdelrazik, Mahmoud Eldesouky, Ibrahim Antoun, Kaung Myat Thu, Akash Mavilakandy, Thet Su, Edward Y. M. Lau, Sherif Altoukhy, Merzaka Lazdam, Zakariyya Vali, Alastair Sandilands, Xin Li, G. André Ng, Mokhtar Ibrahim, Riyaz Somani

**Affiliations:** 1 Department of Cardiology, University Hospitals of Leicester NHS Trust, Glenfield Hospital, Leicester LE3 9QP, UK; ahmed.abdelrazik@leicester.ac.uk (A.A.); mie7@leicester.ac.uk (M.E.); kaung.thu1@nhs.net (K.M.T.); thet.su@uhl-tr.nhs.uk (T.S.); el203@leicester.ac.uk (E.Y.M.L.); sherifaziz_dream@yahoo.com (S.A.); z.vali@leicester.ac.uk (Z.V.); andre.ng@leicester.ac.uk (G.A.N.); mokhtar.ibrahim1@nhs.net (M.I.); riyaz.somani1@nhs.net (R.S.); 2 Division of Cardiovascular Sciences, Clinical Science Wing, University of Leicester, Glenfield Hospital, Leicester LE3 9QP, UK; 3 National Institute for Health Research, Leicester Research Biomedical Centre, Leicester LE3 9QP, UK; 4 Department of Cardiology, Royal Albert Edwards Infirmary, WWL NHS Foundation Trust, Manchester WN1 2NN, UK; am8551995@gmail.com; 5 Department of Engineering, University of Leicester, Leicester LE1 7RH, UK; xin.li@leicester.ac.uk; 6 Leicester British Heart Foundation Centre of Research Excellence, Leicester LE1 7RH, UK

**Keywords:** artificial intelligence, large language model, Wolff–Parkinson–White, accessory pathway, ECG interpretation, electrophysiology

## Abstract

**Background/Objectives:** Accurate localisation of manifest accessory pathways from the 12-lead electrocardiogram remains clinically relevant in Wolff–Parkinson–White syndrome, particularly for pre-procedural planning. Although purpose-built artificial intelligence models have shown promise in ECG interpretation, the reliability of general-purpose multimodal large language models for accessory pathway localisation is unknown. We evaluated two contemporary general-purpose AI systems against an electrophysiology-confirmed reference standard and assessed reproducibility across repeated analyses. **Methods:** In this retrospective, single-centre proof-of-concept diagnostic accuracy study, 49 consecutive patients with manifest accessory pathways confirmed during electrophysiology study/ablation were included. Anonymised pre-procedural 12-lead ECGs were compiled into a single PDF and analysed by ChatGPT 5 Thinking and Gemini 2.5 Pro using predefined EASY-WPW anatomical categories. Each model was tested in three independent context-reset runs. The primary outcome was repeated-run diagnostic accuracy against the electrophysiology-confirmed pathway location, with confidence intervals calculated using an ECG-clustered approach. Secondary outcomes included majority-vote accuracy, pathway-specific descriptive accuracy, exact output consistency, no-consensus outputs, and “unable to identify” responses. **Results:** Each model generated 147 repeated outputs from the same 49 ECGs. ChatGPT 5 Thinking correctly localised 28/147 outputs, corresponding to a repeated-run accuracy of 19.0% (ECG-clustered 95% CI 11.5–26.6), while Gemini 2.5 Pro correctly localised 18/147 outputs, corresponding to 12.2% accuracy (95% CI 6.8–17.7). Both models performed below the no-information majority-class baseline of 36.7%. Majority-vote accuracy was 7/49 for ChatGPT 5 Thinking and 2/49 for Gemini 2.5 Pro. Exact output consistency across all three runs was observed in 2/49 ECGs for ChatGPT 5 Thinking and 0/49 ECGs for Gemini 2.5 Pro. Complete no-consensus outputs occurred in 30/49 and 26/49 ECGs, respectively. “Unable to identify” responses were infrequent: 8/147 outputs for ChatGPT 5 Thinking and 2/147 outputs for Gemini 2.5 Pro. Pathway-specific estimates were descriptive only because of class imbalance and small subgroup denominators. **Conclusions:** General-purpose multimodal large language models demonstrated poor repeated-run accuracy, very low reproducibility, frequent no-consensus outputs, and limited abstention when localising manifest accessory pathways from 12-lead ECGs. These findings do not support their current clinical use for accessory pathway localisation. Future progress is more likely to come from purpose-built, signal-native, or rigorously validated multimodal cardiac AI systems.

## 1. Introduction

Accessory pathways (APs) in Wolff–Parkinson–White (WPW) syndrome are abnormal conduction fibres that bypass the atrioventricular node, thereby pre-exciting the ventricles [1,2]. This can lead to re-entrant tachyarrhythmias and even sudden cardiac arrest in rare cases [2,3]. The 12-lead electrocardiogram (ECG) in manifest WPW classically shows a short PR interval and a delta wave, a slurred upstroke in the QRS complex, indicating ventricular pre-excitation. Identifying WPW syndrome from these ECG features is usually straightforward. However, a critical challenge is localising the exact site of the accessory pathway around the atrioventricular ring using the ECG [4].

Accurate pre-procedural localisation remains clinically relevant because it can help anticipate whether the pathway is left-sided, right-sided, septal, or free-wall, and may inform vascular access, mapping strategy, procedural planning, and expectations regarding technical complexity. This is particularly important for septal pathways, where ablation may be more challenging due to their proximity to the normal conduction system. However, surface ECG localisation is inherently imperfect because the apparent delta-wave vector may be influenced by the pathway insertion site, degree of pre-excitation, atrioventricular conduction balance, cardiac orientation, and lead positioning [5]. Therefore, any tool proposed for accessory pathway localisation must be evaluated not only for diagnostic accuracy, but also for reproducibility and clinical reliability.

Conventionally, clinicians have used stepwise ECG algorithms to localise APs. Starting from the pattern of delta-wave polarity across leads, these decision-tree algorithms classify a WPW ECG into anatomical regions. Examples include the Arruda, Milstein, or D’Avila algorithms from the 1990s [6,7,8]. Recently simplified ECG criteria have shown better performance. For instance, the SMART-WPW algorithm (2025) uses an intuitive clock-face representation of the atrioventricular ring with 12 segments and was prospectively validated in 260 patients [9]. SMART-WPW correctly localised 97% of APs, outperforming older algorithms such as Arruda (which scored ~73% in the same cohort). This demonstrates that even without machine learning, careful re-analysis of ECG features can yield high accuracy. Nonetheless, such algorithms still rely on human interpretation of rules and may not generalise perfectly outside their validation set.

Machine learning and deep learning models can learn complex patterns from large datasets, potentially capturing subtleties that rule-based methods miss. In recent years, AI has achieved notable successes in cardiology, especially with deep neural networks applied to ECG data [10,11]. In the context of WPW syndrome, a study by Nishimori et al. (2021) applied deep learning for AP localisation [12]. Their CNN model, trained on over 200 WPW ECGs, outperformed the conventional decision-tree method, improving accuracy from ~61% to significantly higher values. Interestingly, they further enhanced performance by adopting a multimodal approach that combined the ECG with a patient’s chest X-ray to account for cardiac size and orientation. Another recent effort by Wang et al. (2023) showed that deep learning might even detect concealed accessory pathways from subtle patterns in sinus rhythm ECGs [13], an application traditionally thought impossible with surface ECG alone. These studies underscore the potential of AI: not only can it equal or surpass classical algorithms, but it might also discover new markers of pre-excitation invisible to the human eye.

However, these successful examples largely involve purpose-built ECG AI systems developed for specific electrophysiological tasks. Such models are usually trained or validated using curated datasets, structured reference labels, signal-level ECG data, or task-specific architectures. This is fundamentally different from the use of general-purpose multimodal large language models, which are increasingly accessible to clinicians, researchers, and patients, but are not primarily designed or validated for electrophysiological ECG localisation. When these systems interpret static ECG images or PDF documents, their outputs may depend on document/image processing, inferred visual reasoning, prompt wording, and model stochasticity rather than direct signal-native ECG analysis. This distinction is important because apparent fluency or stepwise reasoning does not necessarily imply diagnostic reliability [14].

Transparent reporting is therefore essential when evaluating AI systems for diagnostic tasks. STARD-AI provides guidance for reporting diagnostic accuracy studies involving artificial intelligence, including a clear description of the dataset, index test, reference standard, evaluation procedures, bias, applicability, and generalizability [15]. DECIDE-AI provides complementary guidance for early-stage evaluation of AI-based decision-support systems, including the intended clinical role, human-AI interaction, safety considerations, and implementation context [16]. Although the present study was a retrospective proof-of-concept diagnostic accuracy evaluation rather than a deployed clinical decision-support intervention, these frameworks are relevant because they highlight the need to assess not only accuracy but also reproducibility, uncertainty handling, and applicability limitations.

Despite progress in both rule-based and machine-learning approaches to accessory pathway localisation, the performance of general-purpose multimodal AI systems in this specific task remains uncertain. This is clinically important because these systems are increasingly accessible but are not primarily designed, trained, or validated for electrophysiological ECG localisation. Their outputs may also vary across repeated prompts because of model stochasticity, interface-level processing, and prompt sensitivity. Therefore, before such systems can be considered for clinical decision support, their diagnostic accuracy, reproducibility, uncertainty handling, and applicability require transparent evaluation. In this retrospective proof-of-concept diagnostic accuracy study, we evaluated two contemporary general-purpose AI systems for localisation of manifest accessory pathways from 12-lead ECGs, using electrophysiology study/ablation findings as the reference standard, and assessed the consistency of their outputs across repeated context-reset analyses.

## 2. Methods

This was a retrospective, single-centre, proof-of-concept diagnostic accuracy study evaluating the ability of general-purpose artificial intelligence systems to localise manifest accessory pathways from standard 12-lead ECGs. Potentially eligible patients were identified from the electrophysiology/catheter laboratory records at the University Hospitals of Leicester NHS Trust, Glenfield Hospital, Leicester, UK, from July 2022 to December 2023. Data collection and AI analysis were performed after completion of routine clinical care and after the electrophysiology study/ablation diagnosis had been established.

Eligible cases were consecutive patients with a manifest accessory pathway on the pre-procedural 12-lead ECG, and a definitive accessory pathway location confirmed at the end of the clinically indicated electrophysiology study/ablation procedure. ECGs were excluded if there was no manifest pre-excitation/accessory pathway on the surface ECG, or if the electrophysiology study did not provide a definitive accessory pathway diagnosis. The final dataset, therefore, comprised 49 consecutive ECGs from patients with electrophysiology-confirmed manifest accessory pathways. The ECG dataset was assembled from routinely acquired clinical 12-lead ECGs and electrophysiology records.

The reference standard was the final accessory pathway location established during the clinically indicated electrophysiology study/ablation procedure. This was selected as the reference standard because invasive electrophysiological mapping and ablation findings represent the clinically definitive method for localising accessory pathways. The accessory pathway location was assigned based on the electrophysiology study/ablation record. The final reference label was based on the clinically documented successful ablation site and procedural electrophysiology interpretation, including mapping findings such as earliest ventricular activation, pathway potential where documented, and electroanatomic or fluoroscopic localisation when available. These procedural locations were then mapped to the predefined EASY-WPW anatomical categories by the study team before AI evaluation. Where the procedural record did not allow definitive assignment to one of the predefined categories, the ECG was excluded from the dataset. The AI systems were not provided with the electrophysiology findings, clinical history or procedural details during ECG interpretation.

We evaluated two contemporary general-purpose AI systems: ChatGPT 5 Thinking and Gemini 2.5 Pro, accessed in October 2025. Both systems were accessed through their standard web-based user interfaces rather than through an application programming interface. The authors did not engineer, train, fine-tune, or externally validate either model. Model temperature and other stochastic generation parameters were not visible or controllable through the interfaces used. The models were evaluated as available to end-users at the time of testing.

Pre-procedural ECGs were anonymised and compiled into a single PDF file. Patient identifiers, hospital numbers, dates of birth, and directly identifiable metadata were removed from the ECG images and PDF file before analysis. No raw digital ECG waveform data, signal-level measurements, clinical notes, procedural reports or electrophysiology labels were provided to the models. The ECGs were interpreted from the uploaded PDF through the native document/image interpretation functionality available within each model interface. The authors did not perform independent optical character recognition, waveform digitisation, signal extraction, or feature engineering before upload. Therefore, the index test reflected multimodal interpretation of static ECG images embedded within a PDF rather than signal-native ECG analysis.

To standardise responses, predefined localisation categories based on the EASY-WPW algorithm were provided: left-sided antero-lateral (LAL), left-sided postero-lateral (LPL), left-sided postero-septal (LPS), right-sided antero-septal (RAS), right-sided postero-septal (RPS), right-sided postero-lateral (RPL), right-sided antero-lateral (RAL), and unable to identify (UN). The anonymised PDF containing all 49 ECGs was uploaded simultaneously, and the following prompt was used: “The attached file contains 49 ECGs for patients with an underlying accessory pathway. Review each ECG individually and identify the location of the accessory pathway. The answer options for each ECG are as follows: Left-sided antero-lateral, left-sided postero-lateral, left-sided postero-septal, right-sided antero-septal, right-sided postero-septal, right-sided postero-lateral, right-sided antero-lateral, unable to identify. Go step by step with detailed reasoning.”

Each AI system was run three times to assess output consistency. A new chat was initiated for each run, and the same anonymised PDF and prompt were provided each time. The models, therefore, had no access to prior answers, preceding outputs, or earlier interpretations from previous runs within the study workflow. The consistency analysis should therefore be interpreted as repeated, context-reset analyses of the same ECG dataset rather than repeated prompting within a continuous conversation. The methodologies described by the AI systems for each run are provided in (Supplementary Material S1) and (Supplementary Material S2).

The primary outcome was overall repeated-run diagnostic accuracy for accessory pathway localisation, defined as the proportion of model outputs that matched the electrophysiology-confirmed accessory pathway location across the three context-reset runs. Because the three outputs for each ECG were repeated predictions from the same case, they were not treated as independent patient-level observations. Overall accuracy was therefore interpreted as the mean repeated-run accuracy across the 49 ECGs, with each ECG contributing up to three model outputs.

Secondary outcomes were pathway-specific accuracy, output consistency across the three repeated runs, the frequency of “unable to identify” responses, and the pattern of reproducibility across repeated outputs. Output consistency was defined as the proportion of ECGs assigned the same pathway category across all three runs. For ECGs with identical outputs across all three runs, classifications were further categorised as consistently correct or consistently incorrect. ECGs for which all three runs produced different pathway categories were classified as no-consensus outputs. Responses of “unable to identify” were permitted as an output option but were treated as incorrect for accuracy analyses and reported separately as abstention events.

Accuracy estimates were reported with 95% confidence intervals. For overall repeated-run accuracy, confidence intervals were calculated using an ECG-clustered approach, with the ECG/patient rather than the individual model output treated as the unit of analysis. Specifically, the number of correct outputs for each ECG across the three runs was converted into an ECG-level repeated-run accuracy value, and confidence intervals were calculated across the 49 ECG-level values. Exact binomial confidence intervals were used for ECG-level binary outcomes, including exact output consistency and majority-vote sensitivity analyses. Output-level confidence intervals for abstention and pathway-specific repeated-run accuracy were reported descriptively.

Because the dataset was imbalanced, with LAL and RPS representing the largest pathway groups, the theoretical uniform chance level of 12.5% was not used as the main inferential benchmark. Instead, model performance was interpreted in relation to the observed class distribution, including the no-information majority-class baseline. In this dataset, the largest pathway category was RPS, comprising 18/49 ECGs; therefore, a classifier always predicting the most common pathway category would achieve an accuracy of 36.7%. Pathway-specific analyses were considered descriptive and hypothesis-generating, particularly where the number of patients within a pathway subgroup was small.

As an ECG-level sensitivity analysis, majority-vote accuracy was also calculated. Where two or more runs were assigned the same pathway category, that category was taken as the model’s majority-vote prediction. Where all three runs produced different pathway categories, this was classified as no consensus and treated as incorrect for the sensitivity analysis.

The AI analysis was performed retrospectively after completion of standard clinical care, and outputs were not used for patient management. No patient-facing interventions occurred, and no AI outputs were returned to clinicians or patients.

Potential sources of bias and applicability were considered during study design and analysis, in line with relevant AI diagnostic accuracy and early-stage AI decision-support reporting principles. These included the retrospective single-centre design, case-selection bias, small sample size, class imbalance, repeated predictions within the same ECG, prompt-induced forced classification, and the use of static ECG images embedded within PDFs rather than raw signal-level ECG data. Consecutive eligible cases were included to reduce selection bias. Repeated outputs were analysed using an ECG-clustered approach, with the ECG/patient rather than the individual model output treated as the unit of analysis, and pathway-specific estimates were treated as descriptive because of small subgroup denominators and class imbalance. Applicability is therefore limited to the specific use case tested: general-purpose multimodal AI systems interpreting anonymised static 12-lead ECG images for manifest accessory pathway localisation using predefined anatomical categories. The findings should not be extrapolated to concealed pathways, raw digital ECG analysis, purpose-built ECG AI models, or prospectively deployed clinical decision-support systems.

## 3. Results

No ECGs were excluded after dataset assembly because of missing model output data. Across the three context-reset runs, all model outputs were available for analysis, giving 147 classifications per model. These outputs represented repeated predictions from the same 49 ECGs and were therefore interpreted as repeated-run outputs rather than independent patient-level observations.

Across the three repeated runs, ChatGPT 5 Thinking correctly localised 28/147 outputs, corresponding to a repeated-run accuracy of 19.0% (ECG-clustered 95% CI 11.5–26.6). Gemini 2.5 Pro correctly localised 18/147 outputs, corresponding to a repeated-run accuracy of 12.2% (ECG-clustered 95% CI 6.8–17.7). These values represent mean repeated-run accuracy across the 49 ECGs rather than accuracy in 147 independent ECGs.

The dataset was imbalanced, with LAL accounting for 17/49 ECGs and RPS for 18/49 ECGs. Therefore, a no-information classifier always predicting the most common class, RPS, would achieve 18/49 accuracy, corresponding to 36.7%. In this context, the theoretical uniform chance level of 12.5% was not considered an appropriate primary inferential comparator. Both models, therefore, performed poorly in absolute terms and below the majority-class baseline.

Output consistency across repeated context-reset runs was very low. ChatGPT 5 Thinking assigned the same pathway category across all three runs for 2/49 ECGs, corresponding to an exact consistency rate of 4.1% (95% CI 0.5–14.0). Of these two ECGs, one was consistently correct and the other consistently incorrect. Gemini 2.5 Pro did not assign the same pathway category across all three runs for any ECG, corresponding to an exact consistency rate of 0/49 (0.0%; 95% CI, 0.0–7.3). Variable classifications were therefore observed in 47/49 ECGs for ChatGPT 5 Thinking and 49/49 ECGs for Gemini 2.5 Pro.

Complete no-consensus outputs, where all three runs produced different pathway categories, occurred in 30/49 ECGs for ChatGPT 5 Thinking and 26/49 ECGs for Gemini 2.5 Pro. As an ECG-level sensitivity analysis, majority-vote accuracy was 7/49 for ChatGPT 5 Thinking, corresponding to 14.3% (95% CI 5.9–27.2), and 2/49 for Gemini 2.5 Pro, corresponding to 4.1% (95% CI 0.5–14.0). These sensitivity findings further support the limited reliability of both models.

The “unable to identify” option was selected 8/147 times by ChatGPT 5 Thinking, corresponding to an abstention rate of 5.4% (95% CI 2.4–10.4), and 2/147 times by Gemini 2.5 Pro, corresponding to an abstention rate of 1.4% (95% CI 0.2–4.8). These responses were treated as incorrect for accuracy analyses but are reported separately because abstention behaviour is clinically relevant when assessing decision-support reliability.

**Figure 1:**
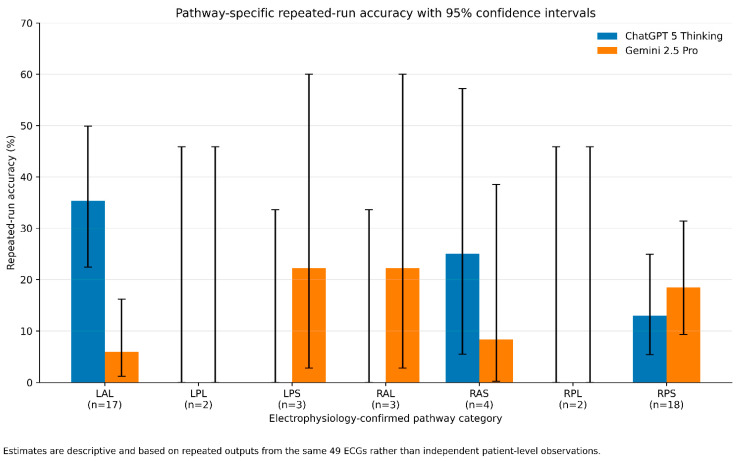
Descriptive pathway-specific repeated-run accuracy of ChatGPT 5 Thinking and Gemini 2.5 Pro for accessory pathway localisation.

Table 1 summarises the overall repeated-run performance, reproducibility, majority-vote sensitivity analysis, and abstention behaviour for both models. Table 2 and Figure 1 present descriptive pathway-specific repeated-run accuracy with 95% confidence intervals; these subgroup estimates were considered hypothesis-generating only because of class imbalance and small pathway-specific denominators.

Pathway-specific findings were descriptive and hypothesis-generating. Several pathway categories had very small numbers of patients, including LPL and RPL with 2 patients each, LPS and RAL with 3 patients each, and RAS with 4 patients. The resulting confidence intervals were wide, and pathway-level *p*-values were therefore not emphasised. Overall, both models demonstrated poor repeated-run accuracy, very low output consistency, limited abstention, and limited practical value for accessory pathway localisation.

Repeated-run accuracy represents the proportion of correct outputs across three context-reset runs of the same 49 ECGs. These 147 outputs were not treated as independent patient-level observations. Confidence intervals for repeated-run accuracy were calculated using an ECG-clustered approach, with the ECG/patient as the unit of analysis. Majority-vote accuracy was included as an ECG-level sensitivity analysis. Exact output consistency was defined as the assignment of the same pathway category across all three runs.

Pathway-specific estimates are descriptive and hypothesis-generating only. They are based on repeated model outputs from the same ECGs rather than independent patient-level observations. Confidence intervals are shown to illustrate statistical uncertainty. Interpretation is particularly limited for pathway categories with very small patient numbers, including LPL and RPL with two patients each, LPS and RAL with three patients each, and RAS with four patients. 

Bars show repeated-run accuracy across three context-reset runs, grouped by the electrophysiology-confirmed pathway category. Error bars represent 95% confidence intervals and are included to illustrate statistical uncertainty. These estimates are based on repeated outputs from the same 49 ECGs rather than independent patient-level observations and should therefore be interpreted descriptively. Pathway-specific interpretation is particularly limited for small subgroups, including LPL and RPL with two patients each, LPS and RAL with three patients each, and RAS with four patients. No pathway-level inferential conclusions were drawn from these subgroup estimates.

## 4. Discussion

In this retrospective proof-of-concept study, two general-purpose multimodal large language models demonstrated poor accuracy and very low reproducibility when asked to localise manifest accessory pathways from 12-lead ECGs. Across three independent context-reset runs of the same 49 ECGs, ChatGPT 5 Thinking achieved a repeated-run accuracy of 19.0%, while Gemini 2.5 Pro achieved 12.2%, both with wide confidence intervals and both below the no-information majority-class baseline of 36.7%. ECG-level majority-vote analysis showed similarly limited performance, with correct localisation in only 7/49 ECGs for ChatGPT 5 Thinking and 2/49 ECGs for Gemini 2.5 Pro. These findings indicate that, in their current off-the-shelf form and when used with static ECG PDFs, general-purpose conversational AI systems are not reliable for accessory pathway localisation or pre-procedural decision support.

These results should not be interpreted as evidence that artificial intelligence cannot assist in this task. Rather, they highlight an important distinction between general-purpose LLMs and purpose-built ECG AI. Prior work has shown that dedicated deep-learning models trained directly on ECG data can outperform conventional rule-based algorithms for WPW localisation, and that multimodal approaches combining ECG with chest radiography can further improve performance. More recent work also suggests that refined structured ECG algorithms such as EASY-WPW and SMART-WPW can achieve clinically useful localisation accuracy when applied in the appropriate setting [17]. In contrast, the models evaluated in the present study were general-purpose systems operating on static ECG images and relying on inferred visual-textual reasoning rather than signal-level learning. That design difference is likely central to the performance gap observed here.

The present findings should therefore be interpreted as a limitation of general-purpose conversational AI for this specific ECG-localisation task, rather than as evidence against AI in electrophysiology more broadly. Contemporary reviews of AI in cardiac electrophysiology describe increasingly successful applications across the clinical pathway, including automated ECG interpretation, arrhythmia detection, electroanatomic mapping, ablation guidance, risk prediction, and treatment personalisation. These applications typically depend on task-specific datasets, structured electrophysiological inputs, signal-native modelling, or integration with procedural mapping systems. This contrasts with the present study, in which the models interpreted static ECG images embedded within a PDF without access to raw waveform data, electroanatomic information, or task-specific training. The performance gap reinforces the need to distinguish between validated electrophysiology-focused AI systems and general-purpose multimodal LLMs used outside their intended or validated domain [18].

A notable finding was that model errors appeared to be patterned rather than purely random. Both systems produced plausible stepwise explanations that echoed established ECG localisation heuristics, including reliance on V1 and inferior lead polarities, as well as the precordial transition. However, these rules were applied inconsistently, and performance remained poor, particularly for septal pathways, which are already recognised as among the most difficult locations to classify accurately from surface ECG alone. The models also showed little tendency to abstain when uncertain, despite being given an “unable to identify” option, suggesting a bias toward forced classification rather than calibrated uncertainty. This combination is clinically important because a model that generates fluent reasoning while arriving at unstable or incorrect conclusions may be more misleading than one that simply fails. More broadly, this is consistent with emerging literature showing that LLM-based ECG interpretation remains constrained by limited signal-native understanding, uncertainty calibration, and reproducibility [19,20].

The low consistency across repeated runs is itself an important negative finding. For any diagnostic support tool, stability is a prerequisite for trust. In the present study, repeated analysis of the same ECGs yielded concordant outputs in only a small minority of cases, suggesting that predictions were strongly influenced by internal stochasticity rather than anchored to a stable interpretation of the tracing. In practical terms, a tool that may give different pathway localisations for the same ECG on separate occasions is difficult to justify in a clinical workflow. This is especially relevant in WPW, where pre-procedural localisation is used to inform the mapping strategy, procedural planning, and expectations for technically challenging sites, such as the septal pathways.

From a clinical perspective, these findings argue against the use of current general-purpose LLMs for accessory pathway localisation in routine practice. At present, established ECG algorithms and expert electrophysiology interpretation remain the appropriate framework for pre-ablation localisation. The more realistic future role of AI in this field is likely to come from domain-specific models trained on raw digital ECG signals, multimodal systems that integrate anatomical information, or approaches that use structured ECG feature extraction rather than interpreting image-based PDFs. Recent WPW-specific AI studies support this direction, as their stronger results were achieved with dedicated architectures, curated datasets, and task-specific development rather than with generic chatbot models [12].

## 5. Limitations

This study was retrospective, single-centre, and relatively small, with 49 cases of manifest WPW and unequal representation across pathway classes. Only manifest pathways were included; therefore, the findings should not be extrapolated to concealed pathways or to more complex substrate patterns. The models were tested using static ECG PDFs rather than raw waveform data, and performance may differ with signal-level input, alternative prompting, or future task-specific fine-tuning. In addition, the study reflects specific model versions evaluated at a single time point, and the capabilities of general-purpose AI systems evolve rapidly. Finally, this was a proof-of-concept diagnostic accuracy study benchmarked against the electrophysiology reference standard; it was not designed to determine whether structured feature prompts, external retrieval, or specialised multimodal foundation models could improve performance.

The prompt design may also have influenced model behaviour. Although an “unable to identify” option was provided, the instruction to review each ECG and provide step-by-step reasoning may have encouraged the models to generate plausible localisation explanations even when visual interpretation was unreliable. This is clinically relevant because fluent reasoning can create an impression of confidence despite low diagnostic accuracy. The low abstention rates observed in this study, despite poor performance, suggest limited calibration of uncertainty and a tendency toward forced classification. We did not perform an additional post hoc conservative-prompt sensitivity analysis because the primary aim was to benchmark a predefined standardised prompt across both systems; introducing a second prompt strategy after the primary analysis would constitute a separate exploratory experiment. Future studies should evaluate whether prompts that explicitly encourage abstention, require uncertainty scoring, or request structured ECG features before final classification improve calibration and reliability.

## 6. Conclusions

In this retrospective proof-of-concept evaluation, general-purpose LLMs demonstrated poor repeated-run accuracy and very low output consistency when localising manifest accessory pathways from 12-lead ECGs, as benchmarked against electrophysiology-confirmed diagnoses. Both models showed limited practical reliability, with low accuracy, poor reproducibility across context-reset runs, frequent no-consensus outputs, and limited abstention despite the availability of an “unable to identify” option. Interpretation of pathway-specific performance was constrained by class imbalance, wide confidence intervals, and small subgroup denominators. These findings do not support the current use of general-purpose conversational AI systems for accessory pathway localisation in clinical practice. Future progress in this area is more likely to come from purpose-built, signal-native or rigorously validated multimodal cardiac AI systems.

## Figures and Tables

**Table 1:** Overall repeated-run performance, reproducibility, and abstention.

Measure	ChatGPT 5 Thinking	Gemini 2.5 Pro
ECGs analysed	49	49
Repeated outputs analysed	147	147
Correct repeated-run outputs	28/147	18/147
Repeated-run accuracy	19.0%	12.2%
ECG-clustered 95% CI for repeated-run accuracy	11.5–26.6	6.8-17.7
Majority-vote accuracy, sensitivity analysis	7/49 (14.3%)	2/49 (4.1%)
95% CI for majority-vote accuracy	5.9–27.2	0.5–14.0
Exact same output across all three runs	2/49 (4.1%)	0/49 (0.0%)
95% CI for exact output consistency	0.5–14.0	0.0–7.3
Consistently correct across all three runs	1/49	0/49
Consistently incorrect across all three runs	1/49	0/49
Variable classifications across runs	47/49	49/49
No-consensus ECGs, all three runs different	30/49	26/49
“Unable to identify” outputs	8/147 (5.4%)	2/147 (1.4%)
95% CI for “unable to identify” outputs	2.4–10.4	0.2–4.8

**Table 2:** Descriptive pathway-specific repeated-run accuracy by true pathway category.

True Pathway	Patients	ChatGPT Correct/Outputs	ChatGPT Accuracy (%)	ChatGPT 95% CI	Gemini Correct/Outputs	Gemini Accuracy (%)	Gemini 95% CI
LAL	17	18/51	35.3	22.4–49.9	3/51	5.9	1.2–16.2
LPL	2	0/6	0	0.0–45.9	0/6	0	0.0–45.9
LPS	3	0/9	0	0.0–33.6	2/9	22.2	2.8–60.0
RAL	3	0/9	0	0.0–33.6	2/9	22.2	2.8–60.0
RAS	4	3/12	25	5.5–57.2	1/12	8.3	0.2–38.5
RPL	2	0/6	0	0.0–45.9	0/6	0	0.0–45.9
RPS	18	7/54	13	5.4–24.9	10/54	18.5	9.3–31.4

## Data Availability

The data related to this study are shared upon reasonable request from the corresponding author.

## Supplementary Materials

The following supporting information can be downloaded at: https://www.mdpi.com/article/10.3390/jcm15114058/s1, Supplementary S1 and Supplementary S2.
