# Supplementary material for: Benchmarking General Purpose Artificial Intelligence for Accessory Pathway Localisation on 12-Lead Electrocardiograms: A Proof-of-Concept Study
Source: J Clin Med. 2026 May 24;15(11):4058. doi: 10.3390/jcm15114058 (PMC13258361; doi:10.3390/jcm15114058)
Supplement: Supplementary file 1 [file jcm-15-04058-s001.zip › Supplementary Material S1.pdf]

ChatGPT-5 thinking:

First run methodology:

Use the most pre-excited beats. If pre-excitation varies, pick the shortest PR / largest delta.

Step 1 — Left vs Right (first pass)

- V1 delta positive → Left-sided. V1 delta negative → Right-sided. (Core rule used in several modern algorithms, including EASY-WPW.)

Step 2 — Anterior vs Posterior (use inferior leads)

- Inferior leads (II/III/aVF) predominantly positive → Anterior.
- Inferior leads predominantly negative → Posterior. (Common across Arruda/derivatives; many algorithms key on inferior delta polarity for ant/post.)

Step 3 — Septal vs Free-wall (use V1/V2 and transition)

- Septal pathways tend to show early precordial transition (≤V2–V3); free-wall tends to transition later (≥V3–V4), with characteristic limb-lead patterns. (Used in classic Arruda/Fitzpatrick-style logic.)

Step 4 — Tie-breakers for posteroseptal vs posterolateral

- Deep negative early delta in lead II favours a left posteroseptal/CS region (subepicardial) vs. posterolateral.
- For right posteroseptal, lead III often more negative than II, and V1 is frequently negative with rapid transition. (Useful discrimination across inferior septal subgroups.)

Putting it together for your 8 answer buckets:

- Left-sided antero-lateral: V1 delta +, inferior +, lateral limb leads (I/aVL) +, transition often ≤V3–V4.
- Left-sided postero-lateral: V1 +, inferior −, I/aVL +; no strong septal features; transition mid-precordial.
- Left-sided postero-septal: V1 + or small; inferior − (esp. II negative very early), septal/CS clues; transition early.
- Right-sided antero-septal: V1 −, inferior + (anterior), early transition (≤V2); I/aVL can be −/isoelectric.
- Right-sided postero-septal: V1 −, inferior − (posterior), III more negative than II helpful; transition ≤V2–V3.

- Right-sided postero-lateral: V1 −, inferior −, later transition (≥V3), I/aVL often −.
- Right-sided antero-lateral: V1 −, inferior +, later transition (free-wall); I/aVL often −.
- Unable to identify: mixed/flat delta or minimal pre-excitation; rate-dependent changes; conflicting signals (e.g., congenital anomalies, multiple APs). Consider using most pre-excited beats or repeat ECG.

These rules are distilled from widely used algorithms (Arruda/Fitzpatrick derivatives and EASY-WPW); I'll cite whichever branch applies as I justify each case.

<u>Second run methodology:</u>

1. Determine side (left vs right) from lead V1 delta-wave polarity

- Δ in V1 positive → Left-sided AP most likely.
- Δ in V1 negative → Right-sided AP most likely.
- Δ in V1 is isoelectric/biphasic → Think septal/paraseptal (will refine with next steps).
  (These are core features in validated algorithms and summaries.)

2. Determine free-wall vs septal using limb leads (lead I/aVL and inferior leads)

- Inferior leads (II, III, aVF):
  • Strongly positive Δ in ≥2 of 3 → anterior half of the annulus.
  • Clearly negative Δ in ≥2 of 3 → posterior half of the annulus.
- Lead I / aVL polarity & magnitude help distinguish left lateral vs septal/posteroseptal (see next step).

3. Refine the exact sector using "most positive delta" (EASY-WPW) and classic Arruda cues.

- For left-sided APs (ΔV1 positive):
  • Most positive Δ in III → Left antero-lateral
  • Most positive Δ in II or aVR → Left postero-lateral
  • Most positive Δ in aVL → Left postero-septal
- For right-sided APs (ΔV1 negative): algorithm extends with lead I/aVR and precordials to separate antero-septal vs antero-lateral vs postero-lateral/postero-septal (I'll reference both EASY-WPW and Arruda patterns during the call).

4. Septal fine-tuning (especially posteroseptal)

- Posteroseptal pathways often show negative Δ in inferior leads and characteristic V1 features; truly negative Δ in lead II raises suspicion for epicardial/CS (coronary

venous) posteroseptal insertion. I'll note when the pattern suggests right- vs left-sided posteroseptal (tricuspid vs mitral annulus).

5. Sanity checks I'll mention in the reasoning

- Degree of pre-excitation (if minimal, morphology can be misleading).
- QRS transition across V1–V3 (late transition favors left free-wall; early transition can favor right free-wall).
- Look for confounders (bundle branch block, baseline axis deviation).

<u>Third run methodology:</u>

1. Pick the beat with maximal pre-excitation (shortest PR / biggest delta).
2. Left vs right side (first pass):
   • V1 delta wave positive → left-sided AP.
   • V1 delta wave negative or isoelectric → right-sided/septal AP.
3. If left-sided (V1 positive): use the most positive delta among leads II, III, aVR, aVL:
   • Most positive in aVL → left postero-septal
   • Most positive in II or aVR → left postero-lateral
   • Most positive in III → left antero-lateral
4. If right-sided (V1 negative/iso):
   • Assess precordial transition (V3) to separate septal vs free-wall, then again use which of II, III, aVR, aVL is most positive to decide among right antero-septal / right postero-septal / right antero-lateral / right postero-lateral (this is the EASY-WPW approach). Accuracy is generally high for free-wall pathways and a bit lower for septal ones.
