# Supplementary material for: Benchmarking General Purpose Artificial Intelligence for Accessory Pathway Localisation on 12-Lead Electrocardiograms: A Proof-of-Concept Study
Source: J Clin Med. 2026 May 24;15(11):4058. doi: 10.3390/jcm15114058 (PMC13258361; doi:10.3390/jcm15114058)
Supplement: Supplementary file 1 [file jcm-15-04058-s001.zip › Supplementary Material S2.pdf]

Gemini 2.5 Pro

First run methodology:

Each ECG is assessed using a systematic approach based on the polarity of the delta wave and the QRS morphology across the 12 leads

Second run methodology:

The localization is primarily based on the polarity of the delta wave (the initial slurring of the QRS complex) in various leads and the overall QRS morphology. The key principles are:

- Inferior Leads (II, III, aVF): A negative or isoelectric delta wave suggests a posterior or posteroseptal pathway location. A positive delta wave suggests an anterior or lateral location.
- Lead V1: A positive delta wave (producing a tall R wave) is highly suggestive of a left-sided pathway. A negative delta wave is suggestive of a right-sided pathway. An isoelectric or biphasic delta wave typically indicates a septal pathway.
- Lead aVL: A negative delta wave is a strong indicator of a left-sided lateral pathway.
- Precordial R/S Transition: An early transition (R wave taller than S wave in V1 or V2) suggests a left posteroseptal pathway. A late transition (S wave persists across the precordium) suggests a right-sided pathway.

Third run methodology:

The localisation is primarily based on the direction (polarity) of the delta wave—the initial slurring of the QRS complex—in key ECG leads.

- Delta Wave in V1: A positive delta wave (upright) suggests a left-sided pathway. A negative delta wave (downward) suggests a right-sided pathway. An isoelectric or biphasic delta wave points to a septal pathway.
- Delta Wave in Inferior Leads (II, III, aVF): A positive delta wave suggests a superior/anterior pathway location. A negative delta wave suggests an inferior/posterior location.
- Delta Wave in Lead aVL: A negative delta wave is a strong indicator of a left lateral pathway.
